# Burning by Röntgen Rays

**Published:** 1898-05

**Authors:** William Rollins


### BURNING BY RÖNTGEN RAYS.

TO THE EDITOR:

SIR,—In the March number, on page 198, a case of burning is mentioned which is attributed to Röntgen rays. Now, if there is any fact well established about Röntgen light it is that it cannot burn. It is almost two years since Mr. Tesla stated that the burning was due to the electric discharge, and that if a screen of aluminum was placed between the tube and the patient and well-grounded no burning could take place. In the *Electrical Review* last year I showed by experiment that severe burns could be produced from a tube which gave no Röntgen light, the vacuum being so high that the current could not pass between the terminals, and no screen being placed between my hand and the tube. The matter has therefore been experimentally proved from both sides, and any person who burns a patient is guilty of malpractice and should be sued.

Yours respectfully,

WILLIAM ROLLINS.

